# Lack of SARS-CoV-2 Viral RNA Detection among a Convenience Sampling of Ohio Wildlife, Companion, and Agricultural Animals, 2020–2021

**DOI:** 10.3390/ani13162554

**Published:** 2023-08-08

**Authors:** Margot Ehrlich, Christopher Madden, Dillon S. McBride, Jacqueline M. Nolting, Devra Huey, Scott Kenney, Qiuhong Wang, Linda J. Saif, Anastasia Vlasova, Patricia Dennis, Dusty Lombardi, Stormy Gibson, Alexis McLaine, Sarah Lauterbach, Page Yaxley, Jenessa A. Winston, Dubraska Diaz-Campos, Risa Pesapane, Mark Flint, Jaylene Flint, Randy Junge, Seth A. Faith, Andrew S. Bowman, Vanessa L. Hale

**Affiliations:** 1 College of Veterinary Medicine, The Ohio State University, Columbus, OH 43210, USA; 2 Veterinary Preventive Medicine, College of Veterinary Medicine, The Ohio State University, Columbus, OH 43210, USAvlasova.1@osu.edu (A.V.);; 3 Center for Food Animal Health, Ohio Agricultural Research and Development Center, College of Food, Agricultural and Environmental Sciences, The Ohio State University, Wooster, OH 44691, USA; 4 Cleveland Metroparks Zoo, Cleveland, OH 44109, USA; 5 Cleveland Metroparks, Cleveland, OH 44144, USA; 6 Ohio Wildlife Center, Powell, OH 43065, USA; 7 Veterinary Clinical Sciences, College of Veterinary Medicine, The Ohio State University, Columbus, OH 43210, USA; 8 Center of Microbiome Science, The Ohio State University, Columbus, OH 43210, USA; 9 School of Environment and Natural Resources, College of Food, Agricultural and Environmental Sciences, The Ohio State University, Columbus, OH 43210, USA; 10 Columbus Zoo & Aquarium, Powell, OH 43065, USA; 11 Infectious Diseases Institute, The Ohio State University, Columbus, OH 43210, USA

**Keywords:** SARS-CoV-2, COVID-19, spillover, surveillance, wildlife, domestic animals, agricultural animals

## Abstract

**Simple Summary:**

SARS-CoV-2, the virus lead to global COVID-19 pandemic, is thought to have an animal origin. From humans, SARS-CoV-2 then transmitted to multiple animal species. Infection transmission between species can lead to reservoir species that harbor the virus and promote its reemergence. Between-species viral transmission can also promote viral mutations that make it more harmful to humans or animals or more transmissible to critical populations (e.g., endangered species, agricultural animals). Characterizing SARS-CoV-2 infections across species became a priority early in the pandemic to help understand transmission and susceptibility. In this study, we tested 792 domestic and wild animals around Ohio between May 2020 and August 2021. We focused on high risk animals including sick animals, animals highly susceptible to SARS-CoV-2 (e.g., cats, primates), and highly congregated animals or those with frequent human contact (e.g., shelters, barns, pets). Of the 34 species we tested, SARS-CoV-2 virus was not detected in any sample. Our sampling was largely conducted prior to the peak of human SARS-CoV-2 in Ohio, and this lack-of-detection does not imply lack of susceptibility or transmission between species. Importantly ongoing surveillance is still critical to predicting and preventing future SARS-CoV-2 infections in humans and animals.

**Abstract:**

Severe acute respiratory syndrome coronavirus 2 (SARS-CoV-2) emerged in humans in late 2019 and spread rapidly, becoming a global pandemic. A zoonotic spillover event from animal to human was identified as the presumed origin. Subsequently, reports began emerging regarding spillback events resulting in SARS-CoV-2 infections in multiple animal species. These events highlighted critical links between animal and human health while also raising concerns about the development of new reservoir hosts and potential viral mutations that could alter the virulence and transmission or evade immune responses. Characterizing susceptibility, prevalence, and transmission between animal species became a priority to help protect animal and human health. In this study, we coalesced a large team of investigators and community partners to surveil for SARS-CoV-2 in domestic and free-ranging animals around Ohio between May 2020 and August 2021. We focused on species with known or predicted susceptibility to SARS-CoV-2 infection, highly congregated or medically compromised animals (e.g., shelters, barns, veterinary hospitals), and animals that had frequent contact with humans (e.g., pets, agricultural animals, zoo animals, or animals in wildlife hospitals). This included free-ranging deer (*n* = 76 individuals), free-ranging mink (*n* = 57), multiple species of bats (*n* = 59), and other wildlife in addition to domestic cats (*n* = 275) and pigs (*n* = 184). In total, we tested 792 individual animals (34 species) via rRT-PCR for SARS-CoV-2 RNA. SARS-CoV-2 viral RNA was not detected in any of the tested animals despite a major peak in human SARS-CoV-2 cases that occurred in Ohio subsequent to the peak of animal samplings. Importantly, we did not test for SARS-CoV-2 antibodies in this study, which limited our ability to assess exposure. While the results of this study were negative, the surveillance effort was critical and remains key to understanding, predicting, and preventing the re-emergence of SARS-CoV-2 in humans or animals.

## 1. Introduction

Severe acute respiratory syndrome coronavirus 2 (SARS-CoV-2) emerged in Wuhan, China, at the end of 2019, and ultimately lead to the coronavirus disease 2019 (COVID-19) pandemic [1]. As of June 2023, there have been over 768 million confirmed cases of COVID-19 worldwide, with over 6.9 million deaths [2]. In Ohio alone, there have been 3.4 million confirmed cases and over 42,000 deaths [3]. Current evidence based on viral sequence homology suggests a zoonotic origin [4,5,6]. 

Many other animal species have since demonstrated natural susceptibility to SARS-CoV-2 following contact with infected humans, including domestic cats, dogs, horses, cows, ferrets, mink, lions, tigers, cougars, snow leopards, Asian small-clawed otters, western lowland gorillas, spotted hyena, binturong, coati, fishing cats, lynx, hippopotamus, Syrian golden hamsters, and Eurasian beavers [7,8,9,10,11,12]. Other species have demonstrated experimental susceptibility to SARS-CoV-2 including domestic cats, striped skunks, dwarf hamsters, tree shrews, fruit bats, raccoons, raccoon dogs, deer mice, rabbits, bushy-tailed woodrats, and several non-human primate species [13,14,15,16,17,18,19,20,21,22,23]. Among susceptible species, cats, ferrets, hamsters, fruit bats, raccoon dogs, white-tailed deer, and deer mice have further demonstrated transmission of the virus to conspecifics [17,18,20,21,24,25,26]. Additional animal species such as dolphins, whales, seals, and sea otters are predicted to be susceptible to SARS-CoV-2 based on having orthologous angiotensin-converting enzyme 2 (ACE2) receptor sequences; however, there are no reports of infections in these species to date [27,28]. 

Susceptibility to and transmission of SARS-CoV-2 within and between a growing list of species raises concerns regarding the development of new reservoir hosts, the re-emergence of COVID-19 from these species, and the increased potential for viral mutations that evade immune response. Mounting evidence around white-tailed deer, for example, indicate that deer could serve as a potential reservoir for SARS-CoV-2. White-tailed deer were predicted to be susceptible to SARS-CoV-2 based on ACE2 receptor sequence modeling [27]. Deer were then found to be experimentally susceptible to SARS-CoV-2 and capable of transmitting the virus to conspecifics vertically and horizontally [29,30,31]. Subsequently, SARS-CoV-2 virus and antibodies were identified in large proportions of free-ranging and farmed white-tailed deer in multiple states, including in Ohio, and in Canada [29,30,32,33,34,35,36]. Antibodies against SARS-CoV-2 are detectable for weeks to months, and serological testing for antibodies is a powerful way to identify prior exposure in susceptible species like white-tailed deer [37,38,39,40,41]. A recent study further suggests possible deer-to-human transmission of SARS-CoV-2 [36]. Additionally, one mule deer in Utah was found to have an active SARS-CoV-2 infection and others possessed antibodies [42]. Mule deer are in the same genus as white-tailed deer and both species are present in the county where the positive animals were found. Hamster-to-human transmission of SARS-CoV-2 has also been reported in Hong Kong pet store workers, and one cat-to-human transmission was recently reported in a veterinarian [15,43,44,45]. These cases highlight the critical need for continued surveillance to identify potential hosts that could harbor and disseminate the virus, drive viral evolution, and re-introduce the virus to human populations or to other wildlife/domestic animals. 

Early in the pandemic, along with rising SARS-CoV-2 prevalence in humans, the objective of this study was to broadly surveil for SARS-CoV-2 in free-ranging animals and animals under human care around Ohio from May 2020 to August 2021. Our goal was to assess the prevalence and transmission risk across a wide range of animal species. We specifically tested for SARS-CoV-2 viral RNA, but did not test for antibodies, which limited our ability to detect prior infection or exposure. We focused on species with known or predicted susceptibility to SARS-CoV-2 infection; animals in high-risk situations (e.g., densely congregated or medically compromised); and animals that had frequent contact with humans or environments shared with human—including companion animals (pets), agricultural animals, wildlife, and animals in zoos or hospitals.

## 2. Materials and Methods

### 2.1. Sample Collection

A total of 792 individual animals were sampled from May 2020 to August 2021. Sampling locations included the following: Ohio Wildlife Center, Columbus Zoo & Aquarium, Ohio State University Veterinary Medical Center, MedVet Hilliard, Columbus Humane, Shelter Outreach Services of Ohio, Ohio county fairs and Ohio metroparks. Private citizens—hunters/trappers—also collected samples for this study during fur-bearer season (mink, raccoon, muskrat, coyote, beaver). At the Ohio Wildlife Center, sick, injured, or abandoned native wildlife that presented for care were sampled at intake. At the Columbus Zoo & Aquarium, zoo animals undergoing routine healthcare exams were sampled. At both the zoo and wildlife center, we focused on sampling species known or suspected to be susceptible to SARS-CoV-2. At the Ohio State University Veterinary Medical Center, domestic cats that presented to the Small Animal Emergency and Critical Care Service were sampled at intake. At MedVet Hilliard, domestic ferrets were sampled as part of their routine healthcare exams. At Columbus Humane and Shelter Outreach Services of Ohio, staff veterinarians sampled domestic cats upon intake or existing resident cats that presented with respiratory illness. At Ohio county fairs, show pigs were semi-randomly selected for sampling in each area of the barn. At the Ohio metroparks, we sampled white-tailed deer harvested as part of a population management program. Samples collected for this study were collected from all regions of Ohio, but the majority of the samples came from the Ohio Department of Natural Resources (DNR) District One (central Ohio, 38% of the samples) and District Three (northeast Ohio, 49% of the samples) (Figure S1).

Depending on species and sampling conditions, nasal, oropharyngeal, choanal, conjunctival, and/or rectal swabs (Fisherbrand™ Synthetic-Tipped Applicators) were collected from each animal. For example, for species identified as high risk for SARS-CoV-2 transmission, including domestic cats and ferrets, oropharyngeal, conjunctival, nasal, and rectal swabs were collected, as feasible. From pigs and deer, nasal swabs were collected. From birds, we collected choanal swabs. From bats, oropharyngeal and/or rectal swabs were collected as fecal sampling has been used in previous studies to monitor for coronaviruses in bats [46]. Swabs were immediately placed in a tube with brain heart infusion broth (BHIB), viral transport media, or RNAlater™. When freezers were not immediately available, samples were chilled on ice packs for up to 12 h or placed into a dewar with liquid nitrogen. All samples were then transferred into a −80 °C freezer where they remained until extraction. A few animals (*n* = 9) were tested on multiple dates since SARS-CoV-2 infection could have occurred between testing dates. These animals were counted as a single individual with multiple sampling dates. Animals from which we collected multiple samples on the same date (e.g., nasal and rectal swabs) were also counted as a single individual.

### 2.2. SARS-CoV-2 Testing

SARS-CoV-2 testing was conducted as described previously using one-step real-time, reverse transcription-PCR (rRT-PCR) [33]. In brief, viral RNA was extracted from BHIB, viral transport media, or RNAlater, and the Charité/Berlin World Health Organization (WHO) assay was used to test for SARS-CoV-2 virus RNA. The WHO assay included an E assay (Integrated DNA Technologies cat. no. 1006804) followed by confirmatory and discriminatory RdRp assays (Integrated DNA Technologies cat. no. 10006805 and 10006806) on any samples positive with the E assay [47]. Primers and probes were purchased as a kit from Integrated DNA technologies with the plasmid positive control containing the full genome of Wuhan-Hu-1 (GenBank: NC_045512.2) [47]. In addition to the plate positive control, we added Xeno Internal Control (Life Technologies cat. no. A29763) to each extraction to ensure the validity of extraction and each PCR reaction. Internal positive controls were confirmed positive for all assays.

## 3. Results

Between May 2020 and August 2021, we sampled a total of 792 individual animals, representing 34 different species, and SARS-CoV-2 viral RNA was not detected in any sample (Table 1, Table S1). Per the Ohio.gov COVID-19 dashboard, human SARS-CoV-2 infection rates in Ohio varied widely during this time, peaking in November and December 2020 at 243,131 and 255,965 cases, respectively (2.10% and 2.17% prevalence) [3] (Figure S2). Despite the human caseload of SARS-CoV-2, and although many of the species tested demonstrated moderate to high susceptibility to SARS-CoV-2, none of the animals included in this study tested positive for SARS-CoV-2 virus. Notably, the majority of samples collected in this study were collected prior to the peak in human cases (Figure S2).

## 4. Discussion

In this study, we tested free-ranging and captive wildlife as well as domestic animals around Ohio for SARS-CoV-2 over a 16-month time period that included a COVID-19 peak in late 2020 and early 2021. Although many of the species we tested—including cats, ferrets, mink, white-tailed deer, and non-human primates—have demonstrated moderate to high susceptibility to SARS-CoV-2 in both natural and experimental settings, our early active surveillance efforts largely preceded the peak in human COVID-19 cases in Ohio and did not yield any SARS-CoV-2 detections. While it is possible that we missed viral shedding windows, infection dynamics can also change over time. For example, the 78 free-ranging deer tested in this study were sampled in October–November 2020 in Ohio DNR District 1 and all were negative. In a related study conducted in January–March 2021 using the same methodology and laboratory, 35% (129 of 360) of the free-ranging deer in a different region of Ohio—DNR District 3, northeastern Ohio—tested positive for SARS-CoV-2 [33]. The 2021 study occurred after the human COVID-19 peak and after deer gun hunting season in Ohio (late November–early January), both of which would have created additional opportunities for direct and indirect contact (e.g., environmental contamination) between humans and deer and may have contributed to the SARS-CoV-2 infections observed in deer in early 2021 but not in the fall of 2020 [48]. Other studies in white-tailed deer demonstrate similarly widespread SARS-CoV-2 infection prevalence (>30%) in deer in other regions, with notable increases in prevalence after the winter 2020 human SARS-CoV-2 case peaks [34]. Deer-to-deer transmission of SARS-CoV-2 has also been reported in experimental and natural settings, and deer-specific mutations in SARS-CoV-2 sequences [29,30,31,32,33,34]. Notably, it took time to detect natural infections in deer. These results could also suggest that it took time for SARS-CoV-2 to accumulate in the environment (e.g., runoff/waterways) [49] or in other wildlife species that could indirectly transmit the virus to deer. Negative results in early surveillance efforts did not preclude a potential public health threat associated with animal reservoir establishment. In fact, the threat of re-emergence from animal hosts has already been realized through mink-, hamster-, cat-, and probable deer-to-human transmission events [36,44,45,50,51]. Together, these studies indicate that SARS-CoV-2 may be transmitting, circulating, and evolving more widely than appreciated amongst wildlife, highlighting the importance of continued surveillance in free-ranging animals. 

Amongst the samples collected in this study, 57 were from free-ranging mink (and one under human care). Mink have been identified as potential reservoirs for SARS-CoV-2 and can transmit the virus back to humans [50,51,52,53]. However, most SARS-CoV-2 sampling efforts have focused on farmed mink rather than free-ranging mink [51]. The few SARS-CoV-2 positive free-ranging mink that have been reported were presumed escapees from nearby farms [52]. However, one study in Spain identified two SARS-CoV-2-positive free-ranging mink that were not linked with farms, and instead suggested that SARS-CoV-2 transmission may have occurred via infectious virus in the environment or waterways [54]. Assessing viral prevalence in free-ranging animals is critical to determining the SARS-CoV-2 reservoir and transmission potential of species like mink, and this Ohio study is the largest sampling effort to date, to our knowledge, in free-ranging mink. This study also represents one of the largest sampling efforts in bats (*n* = 59 individuals), another potential reservoir species for SARS-CoV-2. Our samples included four species of bat (Big brown bat, Silver-haired bat, Tri-colored bat, Eastern red bat) all native to Ohio, and all under care at a wildlife hospital in close contact with humans.

Several rodents, including North American deer mice, beavers, and hamsters, have also shown susceptibility to SARS-CoV-2 [11,15,20,43,55]. Moreover, transmission of the virus from hamsters to multiple pet shop workers and owners has been reported in Hong Kong [44]. Deer mice, and the closely related white-footed mouse, are widespread across Ohio and North America and, like white-tailed deer, have the potential to act a host for SARS-CoV-2 [55]. Limited surveillance in these free-ranging species could mean undetected virus circulation and maintenance in the environment. Although our study included 63 samples from other rodent species (American beaver, capybara, groundhog, muskrat, eastern grey squirrel, eastern fox squirrel, eastern chipmunk, American red squirrel), no samples from deer mice were obtained, highlighting key gaps in our surveillance efforts. Targeted efforts to surveil free-ranging deer mice and white-footed mice will be necessary to evaluate natural SARS-CoV-2 infection and transmission in these species as well as their potential to serve as an intermediate host for other animals like white-tailed deer. Identifying approaches to quantify potential intra- and inter-species interactions in wildlife (e.g., GIS, remote sensing, camera traps, habitat and population modeling) will also be critical to assessing transmission risks [56,57,58].

Besides rodents, SARS-CoV-2 infections have been reported in multiple felid species, and one recent case report highlights a suspected cat-to-human transmission of the virus [13,45,59,60,61,62,63]. Of the 277 felids tested in this study, none were positive. Given that there are well over 58 million domestic cats in households in the United States, and tens of thousands of large cats (e.g., tigers, lions, cheetahs, etc.) under human care in zoos or sanctuaries around the U.S, felines warrant continued assessment for SARS-CoV-2 spillover and spillback risk [64]. 

Overall, the results of this study should be interpreted with caution. First, the animals were only tested for SARS-CoV-2 virus, not antibodies. The window to detect virus by rRT-PCR in an infected animal is relatively short. For example, experimentally infected skunks only shed virus for 5 days [14]; cattle, which have demonstrated minimal susceptibility to SARS-CoV-2, may shed virus for 0–3 days [65,66,67], while cats and white-tailed deer have been reported to shed virus up to 21 days [13,29,68]; although, deer only shed infectious virus for approximately 7 days [29]. rRT-PCR testing outside of the viral shedding window will yield a negative result, but antibody testing can reflect exposures that may have occurred weeks to months earlier [31]. Since we did not conduct serological testing for antibodies in this study, it is possible that animals we tested had previous exposure to SARS-CoV-2 but were not shedding virus at the time of testing. Additionally, while we targeted animals at high risk for infection (e.g., susceptible species, highly congregated animals in county fairs or animal shelters, and animal under human care), we did not specifically target animals within COVID-positive households, potentially lowering our likelihood of detection. As such, we cannot rule out the possibility that we failed to detect susceptible or previously infected animals in this study. 

## 5. Conclusions

SARS-CoV-2 is thought to have originated in an animal and spilled over into humans with subsequent spillback from humans into many other animal species. While SARS-CoV-2 remains primarily a human pathogen, known transmission to and from multiple species raises critical public health concerns and the continued need to identify potential competent, amplifying, or reservoir host species, and routes of transmission. From humans to endangered wildlife, zoo animals, pets and agricultural animals, ongoing surveillance is essential as SARS-CoV-2 variants continue to emerge, creating a dynamic landscape of susceptibility and transmission risks within and between species that could have far-reaching implications on conservation, ecosystem health, food production, the economy, and public health. Establishing effective surveillance systems now and developing approaches to evaluate species interactions will also be critical to mitigate future pandemics.

## Figures and Tables

**Table 1:** **Species in Ohio tested for SARS-CoV-2 (May 2020–August 2021).** A total of 801 samples representing 792 individual animals were tested for SARS-CoV-2 via rRT-PCR between May 2020 and August 2021.

Species (Scientific Name)	Species (Common Name)	Number of Individuals Tested	Provenance (HC = under Human Care; FR = Free-Ranging)
**Felids**			
*Acinonyx jubatus*	Cheetah	1 individual, tested on 2 dates	HC
*Felis catus*	Domestic Cat *	275	pets: *n* = 84; shelter: *n* = 191
**Primates**			
*Lemur catta*	Ringtailed lemur	1	HC
*Pongo pygmaeus*	Bornean Orangutan	1	HC
*Trachypithecus cristatus*	Silvered Leaf Langur	1	HC
*Varecia rubra*	Red ruffed lemur	1	HC
*Mandrillus sphinx*	Mandrill	1	HC
*Colobus guereza*	Guereza Colobus Monkey	1	HC
*Pan paniscus*	Bonobo	1	HC
**Rodents**			
*Castor candensis*	American Beaver	4	FR
*Ondatra zibethicus*	Common Muskrat	36	FR: *n* = 57; HC: *n* = 1
*Sciurus carolinensis*	Eastern Grey Squirrel	11	HC
*Sciurus niger*	Eastern Fox Squirrel	6	HC
*Tamiasciurus hudsonicus*	American Red Squirrel	1	HC
*Hydrochoerus hydrochaeris*	Capybara	1	HC
*Tamias striatus*	Eastern Chipmunk	1	HC
*Marmota monax*	Groundhog/Woodchuck	3	HC
**Bats**			
*Eptesicus fuscus*	Big Brown Bat	55 individuals, 6 tested on two dates	HC
*Lasionycteris noctivagans*	Silver-haired bat	1	HC
*Lasiurus borealis*	Eastern Red Bat	2	HC
*Perimyotis subflavus*	Tricolored Bat	1	HC
**Order Carnivora**			
*Mustela furo*	Domestic Ferret	6	pets
*Neogale vison*	American Mink	58	FR: *n* = 57; HC: *n* = 1
*Potos flavus*	Kinkajou	1	HC
*Mephitis mephitis*	Striped Skunk	24 individuals, 1 tested on 2 dates	HC
*Procyon lotor*	Raccoon	15	FR: *n* = 57; HC: *n* = 1
*Lontra canadensis*	North American River Otter	1	HC
*Canis latrans*	Coyote	1	FR
**Birds**			
*Turdus migratorius*	American Robin	1	HC
*Branta canadensis*	Canada Goose	1	HC
**Other Species**			
*Odocoileus virginianus*	White-tailed deer **	76	FR
*Sus scrofa domesticus*	Domestic Pig	184	agricultural
*Sylvilagus floridanus*	Eastern Cottontail Rabbit	12 individuals, 1 tested on two dates	HC
*Didelphis virginiana*	Virginia Opossum	7	HC
	**Total Individuals**	792	
	**Total samples**	801	

* A subset of these domestic cats are described in more detail (J. Winston, in preparation). ** Note: This does not include Ohio deer described in Hale et al., 2021 [33]. In that study, conducted within the same time frame as the present study, ~35% (129 of 360) of the free-ranging deer sampled in Northeast Ohio (Ohio DNR District 3) were SARS-CoV-2 positive via rRT-PCR. All white-tailed deer sampled in this study were from Ohio DNR District 1.

## Data Availability

The data presented in this study are available in Table S1.

## Supplementary Materials

The following supporting information can be downloaded at: https://www.mdpi.com/article/10.3390/ani13162554/s1, Figure S1: Geographical range of samples based on Ohio Department of Natural Resources (DNR) Districts; Figure S2: Ohio SARS-CoV-2 surveillance in humans and animals (May 2020–August 2021). Human data extracted from Ohio.gov [3]. Table S1: Species and sampling dates for all samples that underwent testing for SARS-CoV-2 viral RNA.
