# Supplementary material for: Lack of SARS-CoV-2 Viral RNA Detection among a Convenience Sampling of Ohio Wildlife, Companion, and Agricultural Animals, 2020–2021
Source: Animals (Basel). 2023 Aug 8;13(16):2554. doi: 10.3390/ani13162554 (PMC10451347; doi:10.3390/ani13162554)
Supplement: Supplementary file 1 [file animals-13-02554-s001.zip › animals-2494598-supplementary.pdf]

animals MDPI

Supplementary Material

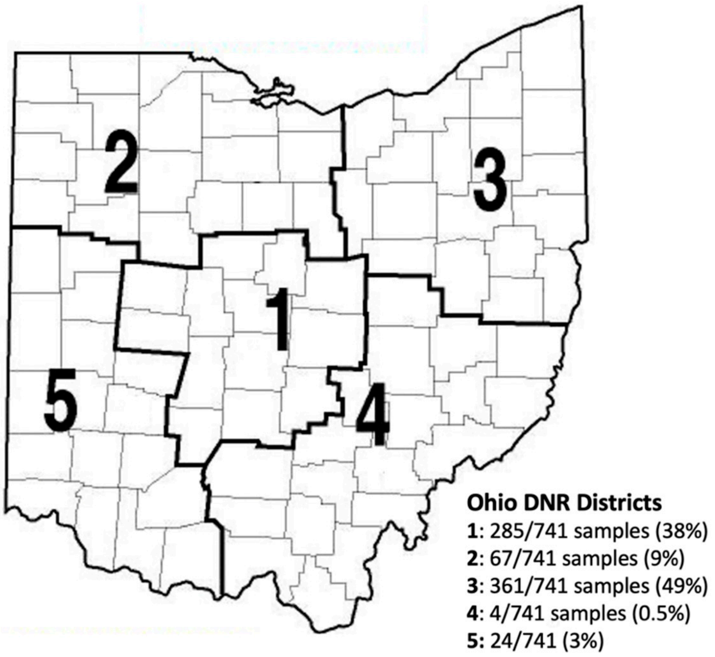

Figure S1. Geographical range of samples based on Ohio Department of Natural Resources (DNR) Districts. Location of origin was only available for 741 of the 801 samples representing 792 individual animals. (Location information was not always recorded for wildlife samples).

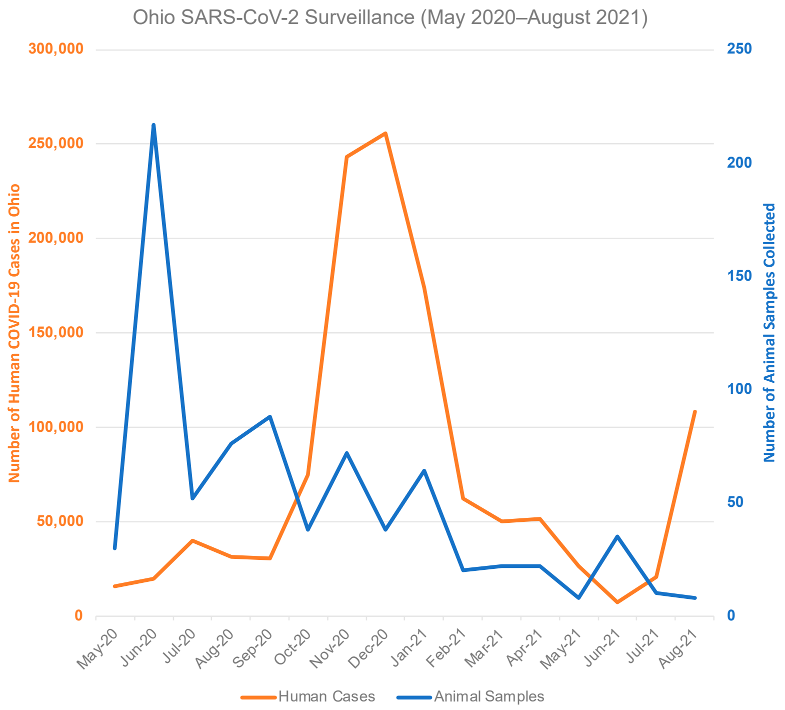

Figure S2. Ohio SARS-CoV-2 surveillance in humans and animals (May 2020–August 2021). Human data extracted from Ohio.gov [3].
